# Transcriptionally Active Chromatin—Lessons Learned from the Chicken Erythrocyte Chromatin Fractionation

**DOI:** 10.3390/cells10061354

**Published:** 2021-05-30

**Authors:** Tasnim H. Beacon, James R. Davie

**Affiliations:** 1 Department of Biochemistry and Medical Genetics, University of Manitoba, Winnipeg, MB R3E 0J9, Canada; beaconth@myumanitoba.ca; 2 CancerCare Manitoba Research Institute, CancerCare Manitoba, Winnipeg, MB R3E 0V9, Canada

**Keywords:** transcriptionally active chromatin, compartment A and B, phase separation, histone modifications, chromatin-modifying enzymes

## Abstract

The chicken erythrocyte model system has been valuable to the study of chromatin structure and function, specifically for genes involved in oxygen transport and the innate immune response. Several seminal features of transcriptionally active chromatin were discovered in this system. Davie and colleagues capitalized on the unique features of the chicken erythrocyte to separate and isolate transcriptionally active chromatin and silenced chromatin, using a powerful native fractionation procedure. Histone modifications, histone variants, atypical nucleosomes (U-shaped nucleosomes) and other chromatin structural features (open chromatin) were identified in these studies. More recently, the transcriptionally active chromosomal domains in the chicken erythrocyte genome were mapped by combining this chromatin fractionation method with next-generation DNA and RNA sequencing. The landscape of histone modifications relative to chromatin structural features in the chicken erythrocyte genome was reported in detail, including the first ever mapping of histone H4 asymmetrically dimethylated at Arg 3 (H4R3me2a) and histone H3 symmetrically dimethylated at Arg 2 (H3R2me2s), which are products of protein arginine methyltransferases (PRMTs) 1 and 5, respectively. PRMT1 is important in the establishment and maintenance of chicken erythrocyte transcriptionally active chromatin.

## 1. Introduction

The chicken erythrocyte is a useful model system to study the organization and function of a vertebrate genome and to discover the salient features of transcriptionally active chromatin [1]. The chicken genome is three times shorter than the human genome but has roughly the same number of genes, with 60% of them with a single human ortholog. The chicken karyotype consists of 38 autosomes and a pair of sex chromosomes (ZW female, ZZ male) and is made up of macrochromosomes (chromosomes 1–8 and Z) and microchromosomes (chromosomes 9–38 and W) [2]. The following features of transcriptionally active chromatin were first documented in studies using chicken erythrocytes: (1) transcriptionally active chromatin is sensitive to DNase I digestion, showing that active chromatin has a “loosened” chromatin structure [3,4]; (2) the boundaries of transcriptionally active chromatin domains (e.g., the β-globin domain) are defined by their DNase I sensitivity [5]; (3) the DNase I sensitivity of transcriptionally active chromatin (typically 2- to 3-fold higher compared to bulk DNA) is increased when the torsional stress of the transcriptionally active chromatin domain is maintained [6,7]; (4) direct demonstration that acetylated histones are associated with the DNase I sensitive transcriptionally active chromatin [8,9].

The chicken red blood cell has an overall compact 30 nm fiber chromatin structure with a higher ratio of linker (H1 and H5) to nucleosomal (H2A, H2B, H3, H4) histones than any other chromatin source [10]. Many sources of chromatin have 0.8 to 1.0 H1 molecules per nucleosome (rat liver, chicken liver, glial nuclei from ox cerebral cortex, pig lymphocytes), while the chicken erythrocyte has 1.3 molecules (0.9 H5, 0.4 H1) per nucleosome. As we will discuss later, histone acetylation, which is limited to transcriptionally active chromatin, prevents H1/H5 from rendering the active gene chromatin insoluble at physiological ionic strength [11]. This composition of the chicken erythrocyte chromatin makes this source of chromatin highly suitable to a chromatin fractionation procedure, allowing the biochemical characterization of a fraction highly enriched in transcriptionally active chromatin.

**Figure 1:**
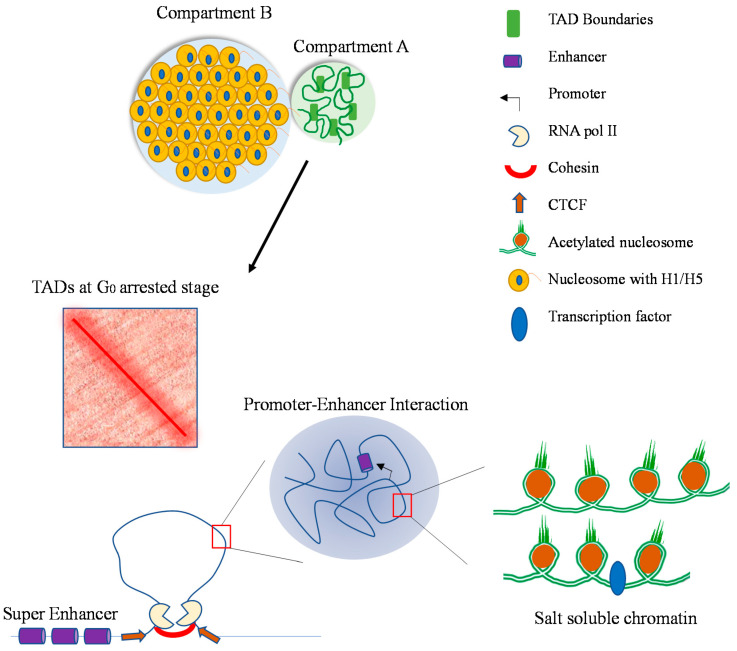
Organization of the chicken erythrocyte genome. Compartment B has condensed chromatin masses organized as 30 nm fibers, which are stabilized by linker histones H1 and H5. Compartment A, at the surface of compartment B, contains transcriptionally active/competent acetylated chromatin, which is associated with the nuclear matrix and is soluble in 150 mM NaCl when released from the matrix.

The chicken erythrocyte genome is organized into compartments (compartment A, transcriptionally active/competent chromatin (1–2%); compartment B, silent, repressed chromatin (98–99%)) [12] (Figure 1). Unlike other vertebrate sources, including chicken fibroblasts, erythrocytes do not have topologically associating domains (TADs) [13]. However, it is possible that TADs are formed with the chromatin in compartment A, which represents a small proportion of the total genome. 

## 2. Enrichment of Transcriptionally Active/Competent Chromatin

**Figure 2:**
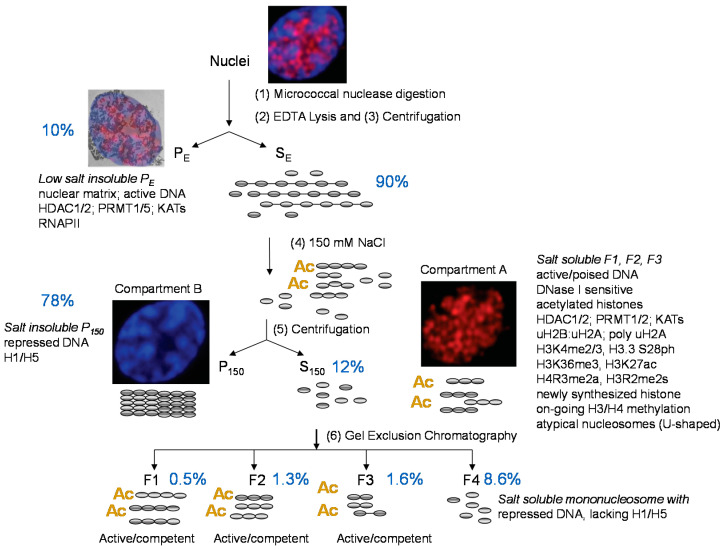
Fractionation of avian erythrocyte chromatin. Chicken polychromatic erythrocyte nuclei were incubated with micrococcal nuclease, and chromatin fragments soluble in a low ionic strength (10 mM EDTA) were recovered in fraction S_E_. Chromatin fraction S_E_ was brought up to 150 mM in NaCl, yielding S_150_ and P_150_. Chromatin fragments from the salt-soluble fraction (S_150_) were size-resolved on a Bio-Gel A-1.5m column to isolate the F1-F3 fractions containing polynucleosomes and oligonucleosomes with active/competent DNA. The composition of the various chromatin fractions is indicated. The percentage of total DNA in each fraction is shown. The low salt-insoluble chromatin fraction P_E_ has the nuclear matrix to which is retained both repressed and transcriptionally active/competent chromatin. Images reproduced with permission from John Wiley and Sons (see Figure 2 in [21]).

When we first tested various chromatin fractionation procedures, we observed that transcriptionally competent β-globin DNA sequences isolated from chicken mature erythrocytes were present in 100 mM KCl-soluble oligonucleosomes, while repressed DNA (e.g., vitellogenin) was in the salt-soluble mononucleosomes [14]. Transcriptionally competent is a term referring to genes that have the DNase I sensitive chromatin structure but are not transcribed. Mature erythrocytes are thought to be transcriptionally silent; however, recent evidence suggests that genes involved in innate immunity can be induced in mature cells [15,16]. To study transcribed genes, we isolate polychromatic erythroid cells from anemic birds. These cells are in the G0 phase of the cell cycle and are transcriptionally active [17]. From lessons learned by testing a variety of chromatin fractionation procedures [18,19], we designed a protocol in which oligo- and polynucleosome length chromatin fragments enriched in expressed genes could be isolated (Figure 2) [20]. The protocol involves micrococcal nuclease digestion of isolated nuclei (step 1) and then lysis of these nuclei in a low-ionic-strength buffer (step 2). Following centrifugation (step 3), the residual low-ionic-strength nuclear pellet (fraction P_E_) and solubilized chromatin (fraction S_E_) are isolated. The fraction P_E_ contains expressed genes in addition to bulk chromatin and chromatin associated with the residual nuclear structure, the nuclear matrix. The S_E_ chromatin is brought up to 150 mM in NaCl (step 4), resulting in the precipitation (fraction P_150_) of most of the chromatin, which is collected by centrifugation (step 5). The soluble chromatin fragments (fraction S_150_) are size-resolved by gel exclusion chromatography (step 6).

Expressed and competent DNA sequences are enriched in the salt-soluble poly- and oligonucleosomes (fractions F1–F3). These fractions are depleted in repressed DNA sequences [22]. The 150 mM NaCl solubility of active genes is proportional to the gene’s chromatin DNase I sensitivity [23]. As we will demonstrate through our analyses of histone post-translational modifications (PTMs), the F1 to F3 chromatin represents active/competent genes in compartment A, while the repressed chromatin in fraction P_150_ is compartment B. Repressed chromatin in faction P_150_ forms 30 nm fibers. This fraction has H1/H5, poorly acetylated histones, uH2A and H4R3me2s (a repressive mark) [22,24]. Repressed DNA sequences are also found in fraction F4, which are mononucleosomes that are soluble in 150 mM NaCl.

The salt fractionation of micrococcal nuclease-generated chromatin fragments has been done with many chromatin sources (calf thymus, Drosophila S2 cells, human HL-60 cells) [19,25,26]. The low salt-soluble (50 to 80 mM NaCl) chromatin fragments enriched in transcriptionally active DNA and acetylated histones are typically of mononucleosome size. In contrast, the chicken erythrocyte 150 mM NaCl-soluble, active DNA sequence-enriched chromatin fragments in F1 to F3 are of poly- and oligonucleosome size.

## 3. Dynamically Acetylated Histones, DNase I Sensitivity and Salt Solubility

Only a small population (1 to 2%) of histones in mature and polychromatic erythrocytes are undergoing dynamic acetylation, and these acetylated histones are associated with the transcriptionally active chromatin fractions [22,27,28]. The four core histones participate in dynamic acetylation, with H2B demonstrating the fastest turnover [27]. Dynamic acetylation is mediated by lysine acetyltransferases (KATs) and histone deacetylases (HDACs). We found that histone acetylation is required for the active gene chromatin to be soluble in 150 mM NaCl. Although the expressed gene chromatin is associated with the linker histones H1 and H5, histone acetylation prevents the active gene chromatin from becoming insoluble at physiological ionic strength [11]. In contrast, linker histones contribute to the insolubility of the poorly acetylated chromatin at physiological strength. The successful performance of this fractionation in chicken erythrocytes relies on their higher level of linker histones compared to other chromatin sources (as mentioned above). To conclude, histone acetylation associated with expressed and competent genes maintains the chromatin in a salt-soluble and DNase I sensitive state.

## 4. Phase Separation

The basis of the chromatin fractionation procedure and the organization of the chicken erythrocyte compartments can be explained in the context of phase separation and a self-organizing system [12]. An important factor in the phase separation of compartment A chromatin (fractions F1–F3 and P_E_) and compartment B (fraction P_150_) is the intrinsically disordered regions in the N-terminal tails of the four core histones and the N- and C-terminal tails of the linker histone H1 and H5 [12,29,30]. The default state of erythroid chromatin is H1/H5-mediated chromatin compaction (Figure 1). Acetylation of the N-terminal tails of the core histones results in a shift from disorder to order [31].

With regard to the steps in the chromatin fractionation protocol, we envision the following occurring simultaneously: (1) micrococcal nuclease incubation of nuclei fragments the chromatin but the compartment A and B remain phase separated; (2) the nuclei are lysed in low ionic strength, phase separation is lost, and compartment A and B chromatin fragments are eluted from the nuclei, yielding fraction S_E_; (3) the S_E_ chromatin solution is brought up to 150 mM NaCl, which results in phase separation of compartment A and B chromatin fragments. Compartment B chromatin fragments form fiber–fiber interactions in a self-organizing system involving unacetylated intrinsically disordered core histone tails and interaction with the intrinsically disordered tails of H1/H5 and separates out of the solution. Compartment A chromatin fragments remain soluble because fiber–fiber interactions are not occurring due to acetylation increasing the ordered α-helical content in the core histone tails. Chromatin fraction P_E_ has both compartment A and compartment B but these also are phase-separated. Compartment A is associated with chromatin-modifying enzymes such as KATs and HDACs, which are associated with the nuclear matrix. The nuclear matrix likely consists of multiprotein complexes including a massive protein–RNA network with the transcriptional machinery, coactivators, transcription factors and RNA, which contributes to the phase separation of compartment A.

The acetylation of the N-terminal tail of H4, particularly at K16, has a major role in decondensation of the chromatin fiber and in phase separation [31,32]. In addition to contributing to the transition from a disordered to an ordered structure, the unacetylated H4 N-terminal tail contacts the nucleosomal DNA at nucleotides 55 and 65 on one strand and nucleotide 88 on the complementary strand [33]. Upon acetylation, this contact is lost, exposing this nucleosomal DNA site to the DNase I nuclease [34]. Further, acetylation of the H3 N-terminal tail at K18, K23 and K27 interacts and alters the structure of the H1 C-terminal tail [35]. Together, these changes in core histone tail–nucleosomal DNA interaction/H1 C-terminal tail structure in acetylated chromatin likely contribute to preventing the default self-associating mode of chromatin compaction and to phase separation.

## 5. F1 DNA Sequences and the Polychromatic Erythrocyte Transcriptome

To identify the DNA sequences enriched in the salt-soluble F1 chromatin, we used next-generation sequencing [2]. The F1 sequences were aligned with RNA sequencing data (polychromatic erythrocyte transcriptome). Highly expressed genes were present in broad salt-soluble chromatin regions. The entire 33-kb β-globin gene domain, which has the highly expressed β-globin (*HBBA*) gene and competent ε-globin (*HBE*) gene, is enriched in F1 chromatin. Other broad F1 domains covering highly expressed genes involved in oxygen carrying and innate immunity functions include α-globin (*HBAA*, 60-kb domain), transferrin receptor (*TFRC*, 35-kb domain), carbonic anhydrase (*CA2*, 86-kb domain), ferritin heavy chain 1 (*FTH1*, 46-kb domain) and interferon-related developmental regulator 1 (*IFRD1*, 33-kb domain). Moderately and low expressing genes (HDAC2 and PRMT7) had mostly the acetylated salt-soluble regions at the upstream promoter region of the gene. With the help of F1 DNA seq, it was possible to map all the salt-soluble acetylated domains across 38 autosomes and the sex chromosomes in female chickens. The genome-wide sequencing revealed that microchromosomes have a higher density of salt-soluble acetylated chromatin than macrochromosomes, implying that gene-dense chromosomes have more salt-soluble chromatin domains.

## 6. Structure of Erythroid Transcriptionally Active Chromatin

The observation that the active gene-enriched chromatin oligonucleosomes presented as a smear rather than a discrete nucleosomal repeat when electrophoretically resolved was a demonstration that these expressed genes have a disrupted chromatin [22,36]. This was further illustrated when analyzed by electron spectroscopic imaging, showing that nucleosomes of the active gene-enriched oligonucleosomes have a U-shaped structure [37]. The U-shaped nucleosomes have 20% less mass than a canonical nucleosome, which is consistent with the loss of an H2A:H2B dimer. This atypical nucleosome structure was first documented by Vincent Allfrey and colleagues, who demonstrated that the U-shaped nucleosome was formed by the elongation of RNA polymerase II [38,39]. Typically, the H3 cysteine sulfhydryl is buried in the nucleosome and not accessible to sulfhydryl reactive compounds. However, in the U-shaped nucleosome, the H3 cysteine sulfhydryl is exposed. Allfrey and colleagues used this feature to isolate these atypical nucleosomes by organo-mercury-agarose column chromatography. The U-shaped nucleosome isolated from chicken erythrocyte chromatin has highly acetylated H4, H3.2 and H3.3. We reported that histone acetylation maintains the U-shaped nucleosome state after being formed by elongation [40].

The mononucleosomes of active chromatin are more labile than canonical nucleosomes. These mononucleosomes were sensitive to ethidium bromide-induced dissociation relative to bulk nucleosomes [23]. Together, these observations suggest that the destabilized U-shaped nucleosomes, once formed, and in a highly acetylated state, would facilitate further rounds of elongation.

The transcriptionally active polychromatic erythrocyte is arrested in the G0 phase of the cell cycle. In the G0 phase, the cells express the replacement histones (e.g., H3.3) rather than the replication-dependent histones. The reassembly of nucleosomes undergoing dissolution during transcription or through the action of chromatin remodelers draws upon the pool of newly synthesized histones. Consistent with this scenario, we found that newly synthesized H2A, H2A.Z, H2B, H3.3 and H4 preferentially exchange with the transcriptionally active chromatin regions [41]. Among these newly synthesized histones, H2A and H2B are prominent in the active chromatin fraction. This is consistent with the U-shaped nucleosome losing and regaining an H2A:H2B dimer. Interestingly, the newly synthesized H2A and H2B in the assembled nucleosome are ubiquitinated, while H2B, H4 and H3.3 are highly acetylated. We assume that these modifications occur after the newly synthesized histones are incorporated into the nucleosome.

Further analyses of the altered structure of the transcriptionally active chromatin were done using hydroxyapatite column chromatography, which showed the increased lability of the H2A:H2B dimer in active chromatin [42]. Interestingly, the H2A variant H2A.Z increases the stability of the H2A H2B in the nucleosome, while uH2A:uH2B dimers destabilized H2A:H2B interactions with the nucleosome, as does acetylation with the histone octamer.

## 7. Histone Post-Translational Modifications and Variants

The ability to isolate transcriptionally active chromatin allowed us to characterize the modified histones and variants associated with transcribed DNA sequences. The acetic acid–urea–Triton X-100 (AUT) PAGE system, which resolves histones according to size, charge and hydrophobicity, was central to the histone analyses [43]. Applying AUT PAGE and SDS PAGE immunoblotting, we showed that the active chromatin fraction F1 from polychromatic erythrocytes is enriched in dynamically acetylated histones, H3R2me2s, H3K4me3, H3S28ph, H3K36me3, uH2B (including di-ubiquitinated H2B), uH2A (including poly-ubiquitinated H2A), H4R3me2a and the variants H2A.Z and H3.3 [20,22,44,45]. The dynamically acetylated H3 and H4 are engaged in ongoing methylation [46]. The H3.3 variant is preferentially phosphorylated at S28, and this modification destabilizes the interaction of H3:H4 tetramer in the nucleosome.

To locate the position of the histone PTMs, we applied the chromatin immunoprecipitation (ChIP) assay and ChIP seq. H3.3 S28ph is present at the promoter region of active genes [45]. As in other vertebrates, H3K4me3 is present at promoters and H3K27ac at the promoters and enhancers of expressed genes [2,24]. As an example, the locus control region of the β-globin domain is marked with nucleosomes containing H3K27ac.

We are the only lab to report the genomic location of H4R3me2a together with H3R2me2s, the products of protein arginine methyltransferase 1 (PRMT1) and PRMT5, respectively [47]. H4R3me2a and H3R2me2s locate largely to introns of expressed genes and intergenic regions, with both marks often co-localizing. H4R3me2a and H3R2me2s are associated with histone marks (H3K4me3) of active promoters, while H4R3me2a co-localizes with H3K27ac at enhancers.

H3K4me3 preferentially locates with CpG islands [48] and, for most genes, is prominent after the first exon [49]. In the rarer distribution, H3K4me3 covers a substantial part of the gene body and regions upstream and downstream of the gene; this is called the broad H3K4me3 domain signature [50,51]. Genes that are essential for the identity and function of a given cell type are marked with a broad H3K4me3 domain that extensively covers the coding region of the gene [50,51,52,53]. We showed that H4R3me2a, H3R2me2s and H3K27ac are associated with broad H3K4me3 domains [47,54].

Transcriptionally competent and active DNA sequences are found in the salt-soluble chromatin fragments (S_150_, F1–F3) and low-ionic-strength insoluble chromatin fraction P_E_. Both F1–F3 and P_E_ chromatin have increased levels of highly acetylated core histones, H3K36me3, H3R2me2s and H4R3me2a, and H3 reactive (U-shaped) nucleosomes. However, one distinguishing feature of F1-F3 is the enrichment in uH2B, which is not observed in P_E_ [22]. We were the first to report that ubiquitination of H2B is dependent on ongoing transcription [55,56]. The transcribed gene body is associated with uH2B [57]. Newly synthesized H2B is ubiquitinated in the salt-soluble chromatin but not in P_E_ chromatin [41]. Together, these observations suggest that the active chromatin that is salt-soluble is enriched in genes undergoing transcriptional elongation.

## 8. Accessible Chromatin and Regulatory Regions

The location of nucleosome-depleted regions (regulatory regions) determined by formaldehyde-assisted isolation of regulatory elements (FAIRE) sequencing was aligned with F1 DNA sequences, ChIP seq data for PTMs and CpG profiling [58]. Analysis of several genes ranging from high to low expression levels (*CA2, FTH1*, β-globin genes, α-globin genes, *ARIH1* and *NCOA4*) showed a similar pattern of feature distribution. Most of these genes have their promoter regions associated with a CpG island. The promoter (nucleosome-depleted region) is marked by a sharp FAIRE seq peak with the F1, H3K4me3 and H3K27ac marks on both sides of the FAIRE seq peak. The enhancer regions were identified in a similar manner. The β-globin domain is a classic example in which the enhancer (LCR) has been identified. The F1 DNA seq signals drop precisely where the FAIRE seq peaks/β-globin enhancers are located. This pattern is typical for most of the genes associated with the transcriptionally active domains. Another example is the erythroid-specific histone H5 gene (*H1F0*). The *H1F0* gene, located in a salt-soluble 48-kb domain [2], is regulated by 5′ and 3′ enhancers. The enhancers are marked with H3K27ac. FAIRE-seq peaks and breaks in the F1 DNA-seq reads (nucleosome-free regions) co-map with these regulatory regions. In vitro and in situ DNase I footprinting showed that the Sp1, GATA-1 and NF1 occupy several binding sites in *H1F0* gene enhancers and promoters in mature and polychromatic erythrocytes [59]. NF1, but not GATA-1 or Sp1, is associated with the nuclear matrix [60].

A small percentage of the genes present an atypical chromatin signature. The chromatin organization of these genes is different from the other genes in the sense that these genes typically do not have a CpG island associated with their promoter and the gene body has broad nucleosome-depleted regions and highly modified (H3K27ac, H3K4me3, H3R2me2s, H4R3me2a) nucleosomes [47,54]. Among these genes are those that are inducible upon external stimuli, e.g., genes involved in the innate immune function (toll-like receptors, interleukins and interferon regulatory factors). Current evidence suggests that the chromatin remodeler (CHD1) is involved in the dissolution and reassembly of nucleosomes associated with the body of genes with this atypical chromatin structure [54].

## 9. Chromatin Modifying Enzymes and Nuclear Location

Dynamic histone acetylation is catalyzed by KATs and HDACs. Fractionation of chicken polychromatic erythrocytes has unveiled the role of the HDACs and KATs in transcriptionally active chromatin. We determined that 70 to 80% of the nuclear HDAC activity was associated with the P_E_ fraction [61]. The ε- and β-globin DNA sequences in this fraction were bound to hyperacetylated H3 and H4 [62]. Further investigation showed that HDAC and KAT activities were associated with the internal nuclear matrix [63,64,65]. Among the HDACs, HDAC1 and HDAC2 were identified as nuclear-matrix-associated HDACs [66,21]. In vitro, the HDAC and KAT enzymes bound to the internal nuclear matrix catalyzed reversible acetylation when endogenous histones of the nuclear matrix-bound chromatin fragments were used as substrates. The chicken erythrocyte HDACs were components of large multimeric complexes, which we assume to be HDAC1/2 in the Sin3, NuRD, Co-REST and RNA splicing proteins [67,68,69,70].

HDAC1 and HDAC2 are phosphorylated by protein kinase CK2 at several sites (HDAC1: S421, S423) (HDAC2: S394, S422, and S424) in the C-terminal part of the protein. This phosphorylation is required for HDAC1/2 to form the Sin3, NuRD and CoREST complexes [71,72,73]. We reported that unmodified and phosphorylated HDAC2 were associated with active chromatin [21,74], but only the phosphorylated HDAC2 was crosslinked to DNA with formaldehyde. To further determine the location of unmodified and phosphorylated HDAC2 in erythrocyte chromatin, we applied two chromatin immunoprecipitation (ChIP) protocols: native (N) ChIP and X-ChIP, which involves cross-linking chromatin with formaldehyde followed by denaturation of chromatin [75]. The N-ChIP assay reveals the genomic location of unmodified and phosphorylated HDAC2, while X-ChIP shows the location of phosphorylated HDAC2. These ChIP assays of dinucleosome containing fraction F2 revealed the association of phosphorylated HDAC2 with regulatory regions of active genes (promoter regions of β^A^-globin and H2A.F), while unmodified HDAC2 was located at the coding region of these genes. We redesigned the X-ChIP protocol to include a protein–protein cross-linking step using the cross-linker dithiobis(succinimidyl propionate) (DSP) [21], which captures both modified and phosphorylated HDAC2. Further, an anti-HDAC2S394ph antibody aided in the location of phosphorylated HDAC2. Similar to our earlier study, HDAC2S394ph was present primarily at the upstream promoter region of the transcribed *CA2* and *GAS41* genes, while unmodified HDAC2 was found within the coding region. The association of HDAC2 to these active genes was partially dependent upon ongoing transcription.

Co-immunoprecipitation studies with RNAPII +/− RNase digestion demonstrated that unmodified HDAC2, but not phosphorylated HDAC2, was associated with RNAPII and that this association was dependent upon the RNA associated with RNAPII. Exploiting the selective cross-linking of phosphorylated HDAC2 to chromatin, we isolated unmodified HDAC2 from fraction F1 chromatin and identified the HDAC2-associated proteins by mass spectrometry. The associated proteins (e.g., SRSF1) were involved in pre-mRNA splicing, a result consistent with the association of unmodified HDAC2 with RNA. HDACs are often found in the spliceosome complexes [70]. Proteins p68, p72 and DEAD-box RNA helicases are associated with class I HDACs (HDAC1, 2 and 3) and play key roles in splicing of pre-mRNAs. The outcome of splicing events depends on the rate of RNAPII elongation and the structure of the chromatin [76]. In the future, to infer splicing events relevant to diseases due to aberrant splicing, machine learning could prove to be a more efficient approach.

Scientists have been testing splicing-directed inhibitors and epigenetic therapy strategies on animal models to treat diseases arising from abnormal splicing events. In chicken, Marek’s disease viruses (MDV) initiate the onset of malignant T-cell lymphomas. All variants of MDVs encode for the Us3 protein kinases, which supports the virus growth. HDAC1 is the common substrate for Us3 for all variants of MDV (1, 2 and 3) (MDV-1 Us3, HDAC1 S406; MDV-2 Us3, S406, S410, S415). MDV Us3 mediates phosphorylation of chicken HDAC1 and HDAC2 (MDV-1 Us3, HDAC2 S407; MDV-2 Us3, S407, S411), regulating their transcription, regulatory interaction and stability. In Liao et al. [77], the authors have shown, using a class I HDAC-specific inhibitor, that the Us3-mediated phosphorylation is essential in the regulation of virus replication, leading to pathogenesis.

As with HDAC1 and HDAC2, PRMT1 and PRMT5 were present in chromatin fractions S_150_ and P_E_ [47]. PRMT1 associates with the transcriptional coactivator CBP/p300, a KAT, which acetylates H3K27 [47]. H4R3me2a stimulates CBP/p300 activity [78], placing PRMT1 upstream of the more well-studied, KAT-dependent gene activation mechanisms. H4R3me2a colocalizes with H3K27ac at intergenic regions and introns [47]. PRMT1 establishes and maintains the transcriptionally active chromatin state [78]. In erythroleukemic cells, silencing PRMT1 precludes the interaction of the multi-enhancer locus control region with the β-globin promoter, reducing globin expression and preventing the formation of active histone PTMs (H3K4 methylation, H3 acetylation) [78,79]. Thus, PRMT1’s activity is a prerequisite to subsequent events required for a functional interaction between the multi-enhancer locus control region and the β-globin promoter and β-globin transcription [78].

## 10. Compartment A and B Location in the Erythroid Nucleus

**Figure 3:**
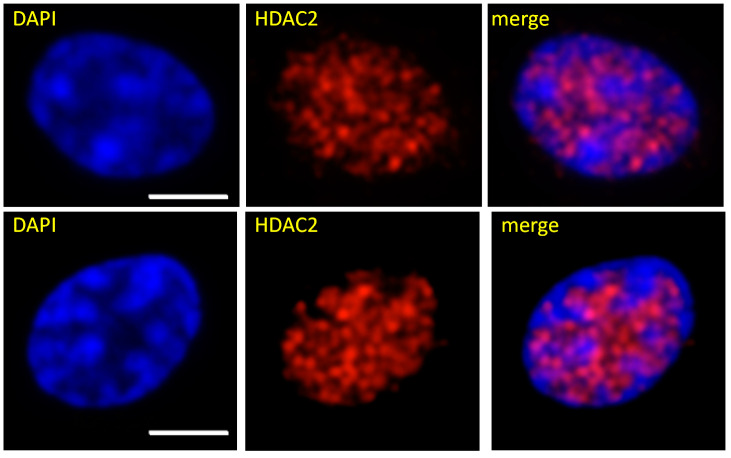
Association of HDAC2 with the interchromatin channels in the chicken polychromatic erythrocyte nucleus. The cells were immunostained with an antibody against HDAC2 and co-stained with DAPI. Spatial distribution was visualized by fluorescence microscopy followed by analyses with AxioVision software. Bar, 5 μm (reprinted with permission from Wiley). Bar, 5 μm. Figure reproduced with permission from John Wiley and Sons (see Figure 2 in [21]).

In terms of compartmentalization, the HDAC2 association with transcriptionally active chromatin has proven to be a probe to compartment A. Cells were immunostained with anti-HDAC2 antibody and co-stained with DAPI. HDAC2, which is primarily bound to the active/competent chromatin in polychromatic erythrocytes, was located in the interchromatin channels, showing the location of compartment A [21] (Figure 3). Compartment B is the more prominent DAPI-stained chromosome masses. Hutchison N and Weintraub H had similar results on visualizing the location of active genes using in situ nuclear nick-translation labelling of chicken red blood cells [80]. DNase I nicks were labelled with biotinyl UTP, which revealed the presence of active genes in the interchromatin channels, further confirming the finding that HDAC2 is highly associated with the transcriptionally active, decondensed chromatin. 

Interestingly, CTCF is also located in the interchromatin channels of chicken erythrocytes [13,81]. Although TADs were not detected in the chicken erythrocyte genome, the compartment A chromatin in the interchromatin channels may be organized in TADs.

## 11. Concluding Remarks

Through use of a powerful chromatin fractionation procedure coupled with histone PTM ChIP seq, FAIRE seq and RNA seq, we have gained detailed information about the genome organization of the chicken erythrocyte. The chicken erythrocyte genome is organized into compartment A (active/competent genes) and compartment B (silent genes). The high levels of linker histones H1 and H5 stabilize the 30 nm fiber organization of compartment B, which presents a compact chromatin in the G0 phase nucleus. Of note, exogenous expression of H5 in cycling rat sarcoma cells resulted in the cessation of replication and arrest in G1. Expression of genes expressed in G1 and differentiation-specific genes did not appear to be affected [82]. In the chicken erythrocyte, compartment A is located in the interchromatin channels at the border of the condensed chromosomes. We envisage that this positioning is achieved by two concurrent forces creating a phase separation between compartments A and B. On one hand, erythroid-specific transcription factors and their roles in attracting coactivators, chromatin-modifying enzymes and remodelers, enhancer–promoter interactions and the transcription machinery play a critical role in placing compartment A chromatin at the periphery of the condensed chromatin masses and in phase separation. On the other hand, the compaction of the compartments B into 30 nm fibers makes way for the formation of interchomatin channels, along with “squeezing out” the compartments A into the channels and concentrating in these channels the factors involved in gene expression and transcriptionally active chromatin structure. These events may be responsible for the formation of the nuclear matrix, which is composed of the transcriptional machinery, coactivators, chromatin-modifying enzymes (KATs, HDACs, PRMTs) and remodelers. The KATs and HDACs are critically important in dynamically acetylating chromatin regions in compartment A as histone acetylation supports a decondensed chromatin structure that is in solution at physiological ionic strength. By the application of predictive models, we will be able to further understand the chicken erythrocyte compartment organization, gaining insight into grouping/clustering by using supervised and semi-supervised techniques.

## Data Availability

No new data were created or analyzed in this study. Data sharing is not applicable to this article.
